# Data suggest COVID-19 affected numbers greatly exceeded detected numbers, in four European countries, as per a delayed SEIQR model

**DOI:** 10.1038/s41598-021-87630-z

**Published:** 2021-04-14

**Authors:** Sankalp Tiwari, C. P. Vyasarayani, Anindya Chatterjee

**Affiliations:** 1 Mechanical Engineering, Indian Institute of Technology Kanpur, Kanpur, 208016 India; 2 Mechanical and Aerospace Engineering, Indian Institute of Technology Hyderabad, Sangareddy, 502285 India

**Keywords:** Applied mathematics, Applied physics

## Abstract

People in many countries are now infected with COVID-19. By now, it is clear that the number of people infected is much greater than the number of reported cases. To estimate the infected but undetected/unreported cases using a mathematical model, we can use a parameter called the probability of quarantining an infected individual. This parameter exists in the time-delayed SEIQR model (Scientific Reports, article number: 3505). Here, two limiting cases of a network of such models are used to estimate the undetected population. The first limit corresponds to the network collapsing onto a single node and is referred to as the mean-$\beta$ model. In the second case, the number of nodes in the network is infinite and results in a continuum model wherein the infectivity is statistically distributed. We use a generalized Pareto distribution to model the infectivity. This distribution has a fat tail and models the presence of super-spreaders that contribute to the disease progression. While both models capture the *detected* numbers well, the predictions of *affected* numbers from the continuum model are more realistic. Our results suggest that affected people outnumber detected people by one to two orders of magnitude in Spain, the UK, Italy, and Germany. Our results are consistent with corresponding trends obtained from published serological studies in Spain, the UK and Italy. The match with limited studies in Germany is poor, possibly because Germany’s partial lockdown approach requires different modeling.

## Introduction

For different countries around the world, several researchers^1–7^ have concluded that the number of people actually infected, or *affected*, by COVID-19 is far greater than the number of cases actually reported, or *detected* officially. Recent serological surveys for COVID-19 also indicate that the infected people outnumber detected people by about 12 times in Spain^8^ and 6 to 24 times in the USA^9^. Other serological surveys suggest that about 18% of people in London^10^ and 23% of the people in New Delhi^11^ were already infected by mid-April and early July, respectively, far outnumbering the reported cases.

In other words, affected numbers seem to greatly exceed detected numbers. To what extent can this difference be anticipated from pure data fitting of detected people, simple parameter estimation, and simple epidemiological models? That is the question we take up in this paper.

We fit two time-delayed SEIQR (Susceptible, Exposed, Infected, Quarantined or Isolated, Recovered/Removed) models to the numbers of reported cases against time, for four European countries. These countries were chosen because they are not extremely large and diverse (e.g., the USA and India), they have cultural differences amongst them, and yet they are geographically close to each other. In other words, they are different from each other but not vastly different. Note that these four countries have largely Caucasian populations, have comparable land areas, have a small latitude and longitude range, allowed free travel between countries prior to the pandemic, and have similar development indices, among other similarities. Yet, there are differences in language, culture, and diet. Moreover, their lockdown policies have been similar in some ways, yet different in others. For instance, Italy, the UK and Spain opted for a full lockdown, while Germany opted for a partial lockdown^12^.

These models are obtained by considering two limiting cases of a time-delayed network SEIQR model motivated by the model of Young et al.^13–15^. In the network model, the whole population is divided into *N* sub-populations based on their net infectivity ($\beta$) values, and each node represents a sub-population or group. In the first limiting case that we adopt, which is the same as a mean-$\beta$ model^15^, the entire network^14^ is collapsed into a single node ($N=1$). This model was originally proposed by Young et al.^13^, and for a fast pandemic some simplifications and approximations are possible^15^. In the second limiting case^14^, which is a continuum model, we take $N \rightarrow \infty$. Here, the infectivity is treated as a continuously distributed parameter in the population. Note that these models are not network models themselves, but are the extreme limits of an underlying network model. Please see the books by Barrat et al.^16^ and Barabási^17^ for more details on network models.

For Italy, Germany, the UK, and Spain, we fit these two models to the data reported under the heading ‘total cases’ on the Worldometer website^18^. We consider the data from February 15–June 18 for fitting (125 days). Beyond mid-June, all the countries seemed to be experiencing a second wave of COVID-19 after relaxing social distancing norms, or perhaps due to increased testing rates. Therefore, the constancy of model parameters beyond mid-June may not be a reasonable assumption.

Both these models include a parameter called the probability of detecting an infected individual. This parameter, upon fitting from detected population data, allows us to indirectly estimate the affected but undetected population. We will find that the continuum model fits the data better than the mean-$\beta$ model. The continuum model predicts that the affected people outnumber the detected people by 8, 22, 48, and 130 times in Spain, the UK, Italy, and Germany, respectively. However, it is emphasized that only the officially detected numbers are used for data fitting. The continuum model also indirectly suggests the presence of ‘super-spreaders’ (or ‘super-spreading events’) in all the countries, in the form of a fat tail in the distribution of the infectivity $\beta$ in the population.

We point out the differences between our network model and complex network models. In our model, each node *k* represents a portion of the susceptible population with infectivity $\beta _k$. Further, each node *k* is connected to every other node $r=1,2,\ldots ,N$ with coupling coefficient $\beta _{kr}=\sqrt{\beta _k}\sqrt{\beta _r}$. In complex network models, these coupling coefficients are referred to as weights^19^. In the continuum limit of our model, when $N \rightarrow \infty$, the initial infectivity corresponding to each node (population group) is assumed to follow a Pareto distribution $\phi (\beta )$.

In complex scale-free networks, the number of nodes are large but finite. Usually, their degree distribution follows a power-law^20^. Sometimes, the distribution of weights of the network may also have a fat tail^19^. Super spreading events are known to occur in complex networks^20^. For example, in the work of Saumell-Mendiola et al.^21^, it was showed that by connecting two complex scale-free networks by a small number of connections (super spreaders), it is possible to achieve endemic equilibrium. Without this small number of connections, the disease did not progress in either of their networks. Finally, we emphasize that our network model’s continuum limit allowed us to make certain mathematical simplifications^14^, which led to a collapse in the dimensionality of the system.

The rest of this paper is organized as follows. In “SEIQR models” section, we briefly discuss the two models (mean-$\beta$ and continuum) used in this work. “The case of Italy” section, we present and discuss in detail the results of the optimization calculations (i.e., parameter fitting) for Italy. In “The cases of Germany, UK, and Spain” section, we present the results for the remaining three countries: Germany, the UK and Spain. In “Conclusions” section, we present our conclusions. In the supplementary material for this paper, we present a detailed study investigating other infectivity distributions in the continuum model, and some sensitivity analysis results.

## SEIQR models

A detailed description of the mean-$\beta$ model^15^ ($N=1$) and the continuum model^14^ ($N\rightarrow \infty$) can be found in the literature. In this section, we describe them briefly for clarity and completeness.

### Mean-$\beta$ model

The mean-$\beta$ model^15^ can be derived from the five state SEIQR model of Young et al.^13^ by assuming no loss of immunity after recovery. This assumption is valid for a fast pandemic like COVID-19. In the mean-$\beta$ model, exposed ($E_m$), quarantined ($Q_m$), and recovered ($R_m$) states becomes slave variables of susceptible ($S_m$) and infected ($I_m$) states whose dynamics are governed by the following DDEs:

1 $$\begin{aligned} {\dot{S}}_m(t)&=-\beta _m S_m(t) I_m(t), \end{aligned}$$

2 $$\begin{aligned} {\dot{I}}_m(t)&=\beta _m S_m(t-\sigma _m) I_m(t-\sigma _m) - p_m e^{-\gamma _m\tau _m}\beta _m S_m(t-\sigma _m-\tau _m) I_m(t-\sigma _m-\tau _m)-\gamma _m I_m(t). \end{aligned}$$

The parameters $p_m$, $\gamma _m$, $\tau _m$, $\beta _m$, and $\sigma _m$ are described in Table 1. The subscript *m* in all the quantities serves to distinguish them from those used in the continuum model ($N\rightarrow \infty$). By defining

3 $$\begin{aligned} V(t)=\int _{-\infty }^t I_m(\eta )\, d\eta , \end{aligned}$$

and integrating Eq. (1), we get

4 $$\begin{aligned} S_m(t)=e^{-\beta _m V(t)}, \end{aligned}$$

where we have imposed the initial condition $S_m(-\infty )=1$. Inserting Eqs. (3) and (4) into (2), we obtain

5 $$\begin{aligned} {\ddot{V}}(t)=\beta _m e^{-\beta _m V(t-\sigma _m)}{\dot{V}}(t-\sigma _m)-p_me^{-\gamma _m\tau _m}\beta _m e^{-\beta _m V(t-\sigma _m-\tau _m)}{\dot{V}}(t-\sigma _m-\tau _m)-\gamma _m {\dot{V}}(t). \end{aligned}$$

Integrating both sides of the above equation and by defining

6 $$\begin{aligned} {\bar{p}}_m=p_me^{-\gamma _m\tau _m}, \end{aligned}$$

we obtain

7 $$\begin{aligned} {\dot{V}}(t)={\bar{p}}_me^{-\beta _m V(t-\sigma _m-\tau _m)}-e^{-\beta _m V(t-\sigma _m)}-\gamma _m V(t) + 1-{\bar{p}}_m. \end{aligned}$$

The complete dynamics of the pandemic in the mean-$\beta$ can be captured by the first-order nonlinear DDE given by Eq. (7). The percentage of population detected as having contracted the disease is given by

8 $$\begin{aligned} h_m(t)=100 {\bar{p}}_m \beta _m \int _{-\infty }^{t} e^{-\beta _m V(t-\sigma _m -\tau _m)} {\dot{V}}(t-\sigma _m-\tau _m) \, dt, \end{aligned}$$

and the percentage of population infected (detected plus undetected) till time *t* is

9 $$\begin{aligned} w_m(t)=100 (1-e^{-\beta _m V(t)}), \end{aligned}$$

The biological parameters $\sigma _m$ and $\gamma _m$ are fixed at values reported in the COVID-19 literature^22–24^.

**Table 1:** Parameters used in the mean-$\beta$ model.

S. no.	Parameter	Description	Ranges	Specified/estimated
1	$\sigma _m$	Asymptomatic and non-infectious period	$\sigma _m=3$	Specified
2	$\tau _m$	Infectious but asymptomatic period	$1 \le \tau_m \le 14$	Estimated
3	$\gamma _m$	Self-recovery rate	$\gamma _m =0.07$	Specified
4	$p_m$	Probability of quarantining symptomatics	$0 \le p_m \le 1$	Estimated
5	$\beta _m$	Infectivity constant	$\beta _m>0$	Estimated
6	$V_0$	History of *V* for DDE	$V_0 >0$	Estimated

### Continuum model

The other limit of the network model^14^ is for the case of $N\rightarrow \infty$, which implies that the infectivity ($\beta$) is now distributed continuously over the population. The governing differential equations for the states *S* and *I* in this case are as follows:

10 $$\begin{aligned} {\dot{S}}(\beta ,t)&=-\sqrt{\beta } S(\beta ,t) \int _0^{\infty } \sqrt{\xi } I(\xi ,t)\, d\xi , \end{aligned}$$

11 $$\begin{aligned} {\dot{I}}(\beta ,t)&=\sqrt{\beta } S(\beta ,t-\sigma ) \int _0^{\infty } \sqrt{\xi } I(\xi ,t-\sigma )\, d\xi -pe^{-\gamma \tau }\sqrt{\beta } S(\beta ,t-\sigma -\tau ) \int _0^{\infty } \sqrt{\xi } I(\xi ,t-\sigma -\tau )\, d\xi -\gamma I(\beta ,t), \end{aligned}$$

where *p*, $\gamma$, $\tau$, and $\sigma$ are described in Table 2. It has been shown^14^ that $S(\beta ,t)$ admits a solution of the form:

12 $$\begin{aligned} S(\beta ,t)=\phi (\beta ) e^{-f(t)\sqrt{\beta }}. \end{aligned}$$

Therefore, if the initial distribution of infectivity in the population, $\phi (\beta )$, is specified, the subsequent variation of *S* is simply through *f*(*t*). Using algebraic manipulations, it has been shown that *f*(*t*) satisfies the following non-linear DDE^14^ :

13 $$\begin{aligned} {\dot{f}}(t)=-G(f(t-\sigma ))+p e^{-\gamma \tau }G(f(t-\sigma -\tau ))-\gamma f(t) + C_0, \end{aligned}$$

where

14 $$\begin{aligned} G(f(t))=\int _0^{\infty } \sqrt{\beta } \phi (\beta ) e^{-f(t)\sqrt{\beta }}\, d\beta , \end{aligned}$$

and

$$\begin{aligned} C_0=(1-pe^{-\gamma \tau })\int _0^{\infty }\sqrt{\beta }\phi (\beta )\, d\beta . \end{aligned}$$

It is useful to introduce a new random variable $u=\sqrt{\beta }$ with probability density function $\psi (u)$, such that $\psi (u)=2u\phi (u^2)$. Then,

15 $$\begin{aligned} G(f(t))=\int _0^{\infty } u \psi (u) e^{-f(t)u}\, du \end{aligned}$$

and

16 $$\begin{aligned} C_0=(1-pe^{-\gamma \tau })\int _0^{\infty } u \psi (u) \, du. \end{aligned}$$

The quantities corresponding to Eqs. (8) and (9) now are

17 $$\begin{aligned} \begin{aligned} h(t)=100pe^{-\gamma \tau } \int _{-\infty }^{t} {\dot{f}}({\bar{t}}-\sigma -\tau ) G(f({\bar{t}}-\sigma -\tau )) \, d{\bar{t}} \end{aligned} \end{aligned}$$

and

18 $$\begin{aligned} \begin{aligned} w(t)=100\left( 1-\int _0^{\infty }S(\beta ,t) \, d\beta \right) =100\left( 1-\int _0^{\infty } \psi (u) e^{-f(t)u} \, du \right) , \end{aligned} \end{aligned}$$

In the present work, we assume $\psi (u)$ to be of the form

$$\begin{aligned} \psi (u)=\frac{(m-1)a^{m-1}}{(a+u)^m}, \quad u > 0, \nonumber \end{aligned}$$

which is a generalized Pareto distribution^25^. This means that

19 $$\begin{aligned} \phi (\beta )=\frac{(m-1)a^{m-1}}{2\sqrt{\beta }(a+\sqrt{\beta })^m}, \quad \beta > 0. \end{aligned}$$

Note that

$$\begin{aligned} \int _{0}^{\infty } \phi (\beta )\, d\beta =\int _{0}^{\infty } \psi (u)\, du=1. \end{aligned}$$

In the supplementary material, we have presented a detailed study investigating other distributions for $\psi (u)$. That discussion supports our choice of the generalized Pareto distribution.

A summary of the parameters used in the continuum model is presented in Table 2. Note that $m>2$ ensures that *u* has a finite mean. If $m>3$, then *u* has finite variance as well.

**Table 2:** Parameters used in the continuum model.

S. No.	Parameter	Description	Ranges	Specified/estimated
1	$\sigma$	Asymptomatic and non-infectious period	$\sigma =3$	Specified
2	$\tau$	Infectious but asymptomatic period	$1 \le \tau \le$ 14	Estimated
3	$\gamma$	Self-recovery rate	$\gamma =0.07$	Specified
4	*p*	Probability of quarantining symptomatics	$0 \le p \le 1$	Estimated
5	*a*	Parameter in $\psi (u)$	$a>0$	Estimated
6	*m*	Denominator exponent in $\psi (u)$	$m > 2$	Estimated
7	$f_0$	History of *f* for DDE	$f_0 > 0$	Estimated

### The fitting error

In the mean-$\beta$ model, for a given set of parameter values, we compute $h_m(t)$ and fit it with the data for the total number of detected cases as reported on the Worldometer website^18^. This is done by minimizing the fitting error

20 $$\begin{aligned} E_{0m}=\frac{||h_m - \text{ data }||_2}{||\text{ data }||_2}\times 100. \end{aligned}$$

We see from Table 1 that there are four parameters to be identified in the mean-$\beta$ model. The fitting error for the continuum model is defined as

21 $$\begin{aligned} E_{0}=\frac{||h - \text{ data }||_2}{||\text{ data }||_2}\times 100. \end{aligned}$$

We see from Table 2 that there are five parameters to be identified in the continuum model.

### Sensitivity analysis

To test the sensitivity of both the models to both fixed and fitted parameters, the parameters are varied by $\pm 2\%$ around the values corresponding to the optimum fit. We generate 10,000 parameter sets using the Latin Hypercube sampling command lhsdesign in MATLAB^26^. Including the two externally specified parameters $\sigma$ and $\gamma$, the hypercube is six dimensional in the mean-$\beta$ model, and seven dimensional in the continuum model. The fits corresponding to these samples are plotted using bands of lighter shades around the optimum fits plotted in darker shades. Results will be presented in "The case of Italy" and "[Sec Sec8]" sections below.

We also study the sensitivity of the fitted parameters to the externally specified parameters $\sigma$ and $\gamma$ by varying them by $\pm 2\%$ around $\sigma =3$ and $\gamma =0.07$. The results of this latter analysis are presented in the supplementary material.

## The case of Italy

The data for the detected cases match very well for Italy. In this section, we present detailed results for Italy obtained from the two models. The results for other countries will be presented in the next section.

### Results for the mean-$\beta$ model

We minimize the fitting error $E_{0m}$ (see Eq. (20)) using the optimization routine fminsearch in MATLAB. Since there are four free parameters in the mean-$\beta$ model, the input variable for the optimization code is a four-by-one column vector, suitably transformed so that the constraints in Table 1 are automatically satisfied. We have performed several hundred optimization calculations with random initial conditions and have found many converging solutions. Several of these solutions correspond to nearly identical and low values of $E_{0m}$. Several other local minima yielded significantly higher $E_{0m}$ values, and were discarded.

**Figure 1:**
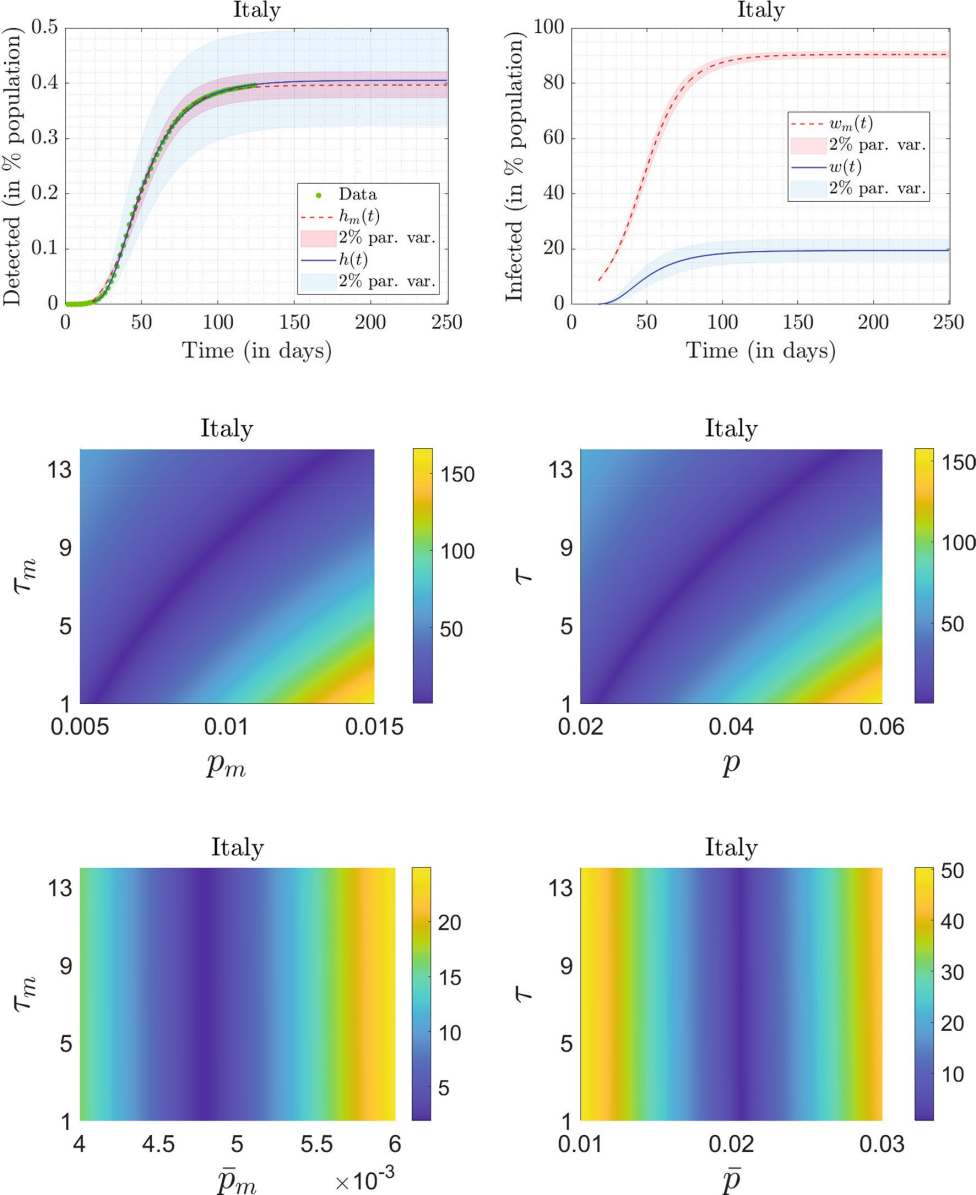
Top row, left panel: fitted results for Italy, $h_m(250)=0.3966\%$ and $h(250)=0.4051\%$. Data in percentage of population for detected cases, obtained from Worldometer, is plotted using green circles. We have plotted the data of alternate days for clarity. The fit to the detected cases obtained using the mean-$\beta$ model is shown by a dashed red curve, and that using the continuum model is shown by a solid blue curve. The parameters used in the mean-$\beta$ model and continuum model for obtaining the fit are reported in row 1 of Tables 3 and 4, respectively. The red and blue shaded bands correspond to $\pm 2\%$ variations in the parameters in the mean-$\beta$ model and the continuum model, respectively. Top row, right panel: Percentage of infected people obtained from the mean-$\beta$ model (dashed red curve) and the continuum model (solid blue curve), respectively; $w_m(250)=90.4331\%$ and $w(250)=19.4350\%$. The red and blue shaded bands correspond to $\pm 2\%$ variations in the parameters in the mean-$\beta$ and the continuum model, respectively. Middle row, left panel: Variation of $E_{0m}$ in the $p_m-\tau _m$ plane (for low values of $p_m$) obtained using the mean-$\beta$ model. The parameters $\beta _m$ and $V_0$ are fixed at the values reported in row 1 of Table 3. Bottom row, left panel: Variation of $E_{0m}$ in the ${\bar{p}}_m-\tau _m$ plane. Middle row, right panel: Variation of $E_{0}$ in the $p-\tau$ plane (for low values of *p*) obtained from the continuum model. The parameters *a*, *m*, and $f_0$ are fixed at the values reported in row 1 of Table 4. Bottom row, right panel: Variation of $E_{0}$ in the ${\bar{p}}-\tau$ plane.

The parameter set that yields the lowest $E_{0m}$ in all the random trials is reported in the first row of Table 3. The fit generated using these parameters, along with the reported data, is shown in the top-left panel of Fig. 1. The reported data, which records the percentage of detected cases in Italy from February 15 for the following 125 days, is plotted in green circles. For easier visibility, only the data of alternate days is plotted. To account for the initial uncertainty in the reporting, we neglect initial data where the number of cases is less than $1\%$ of the number reported on the $125{\text {th}}$ day. The fitted $h_m(t)$ is plotted for a longer duration using a dashed line to depict the saturation value clearly. In the figure, the percentage of detected cases saturates at $0.3966\%$ of Italy’s population.

**Table 3:** Parameter sets from mean-$\beta$ model yielding the lowest $E_{0m}$, and subsidiary quantities.

Country	$\beta _m$	$p_m$	$\tau _m$	${\bar{p}}_m=p_m e^{-\gamma _m\tau _m}$	$V_0$	$E_{0m}$	*A*/*D*	$R_0$
Italy	0.1825	0.0112	12.1157	0.0048	0.4861	1.8770	228	2.5953
Germany	0.2097	0.0069	13.8161	0.0026	0.5211	2.6831	426	2.9882
UK	0.1636	0.0137	12.2077	0.0058	0.4773	1.6211	185	2.3240
Spain	0.1785	0.0194	12.5517	0.0081	0.7386	2.2480	142	2.5296

We also plot the percentage of infected population ($w_m(t)$) in the top-right panel of Fig. 1 with a dashed curve. From the mean-$\beta$ model, the percentage of infected (affected) people early during the progression of the pandemic is around $9\%$, while the saturation value is $90.4331\%$. Both these numbers seem too high, as will be discussed further below.

For this same model, the ratio of the population affected to population detected ($A/D=w_m/h_m$) saturates at 228. The basic reproduction number^13^ $R_0$, for the mean-$\beta$ model, is found from fitted parameters to be

22 $$\begin{aligned} R_0= \beta _m \left( \frac{1-{\bar{p}}_m}{\gamma _m}\right) =2.5953. \end{aligned}$$

The mean-$\beta$ model does offer some further useful insights into data fits, as follows. Upon inspection of the local minima obtained from the fminsearch runs, we noted that all the minima corresponding to low values of $E_{0m}$ have nearly identical $\beta _m$ and $V_0$ (equal to the values reported in the first row of Table 3), but different values for $p_m$ and $\tau _m$. The fitted values of $p_m$ were consistently low, however. To investigate further, we fix the values of $\beta _m$ and $V_0$, and plot $E_{0m}$ in the $p_m-\tau _m$ plane, in the mid-left panel of Fig. 1, for low values of $p_m$. We see that the lowest values for $E_{0m}$ are obtained on a thin band cutting across the $p_m-\tau _m$ plane, which spans the entire assumed range of $\tau _m$ and a relatively much smaller range of $p_m$. Finally, upon plotting $E_{0m}$ in the ${\bar{p}}_m-\tau _m$ plane in the bottom-left panel of Fig. 1, we observe that the thin band corresponds to almost fixed value of ${\bar{p}}_m \approx 0.0048$, indicating that ${\bar{p}}_m$ can be robustly identified, along with $\beta _m$ and $V_0$. In contrast, $\tau _m$ is essentially indeterminate. We now consider the continuum model.

### Results for the continuum model

**Figure 2:**
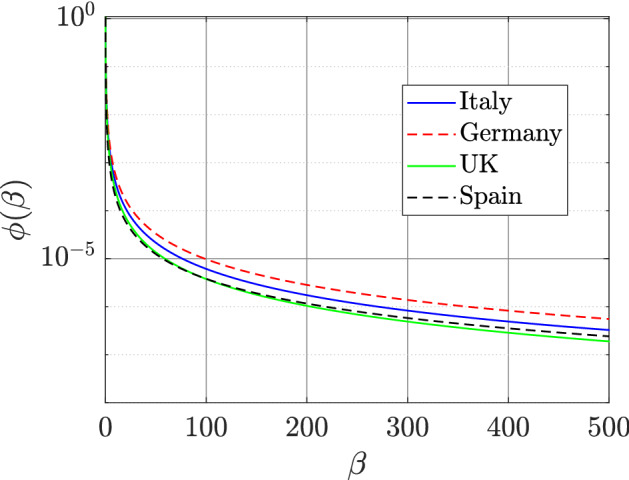
Plots of the fat-tailed distribution ($\phi (\beta )$) as used in the continuum model for Italy, Germany, the UK, and Spain.

In this case, there are five free parameters to be estimated. Therefore, the input variable to the optimization code is a $5 \times 1$ vector, suitably transformed so that the constraints in Table 2 are automatically satisfied. The parameter set that results in the lowest value of $E_{0}$ for all the optimization trials is reported in the first row of Table 4. The corresponding fit *h*(*t*), again plotted for a longer duration to show saturation, is plotted using a solid line in the top-left panel of Fig. 1. The figure indicates that the fit to the detected data from the continuum model is slightly better than that from the mean-$\beta$ model (numerically, $E_{0}=0.6800$ for the continuum model, while $E_{m0}=1.8770$ for the mean-$\beta$ model). The optimizing value of *m* is found to be 2.6688 and corresponds to a fat tail in $\phi (\beta )$’s distribution as shown in Fig. 2. The percentage of detected cases saturate at $0.4051\%$, which is only slightly more than that predicted by the mean-$\beta$ model. We also note that a variation of $\pm 2\%$ in the parameters (both externally specified and fitted) changes this number by about $20\%$, as opposed to about $7\%$ in the mean-$\beta$ model. This means that the continuum model is more sensitive to small changes in parameters, and hence, the fitted parameters are somewhat more robustly determined, as compared to the mean-$\beta$ model.

**Table 4:** Parameter sets from continuum model yielding the lowest value of $E_{0}$ and subsidiary quantities.

Country	*a*	*m*	*p*	$\tau$	${\bar{p}}=pe^{-\gamma \tau }$	$f_0$	$E_0$	*A*/*D*
Italy	0.1351	2.6688	0.0223	1.0659	0.0207	0.0020	0.6800	48
Germany	0.1503	2.5882	0.0199	13.6947	0.0076	0.0008	1.0597	130
UK	0.1185	2.7392	0.0537	2.5073	0.0450	0.0048	0.8896	22
Spain	0.0527	2.4357	0.1768	5.7101	0.1186	0.0002	1.2844	8

When comparing the continuum model with the mean beta model, a large difference is seen in the estimated number of affected people. We plot *w*(*t*) in the top-right panel of Fig. 1 using a solid line. The continuum model predicts that the percentage of population infected, or affected, in Italy saturates at $19.4350\%$, with an affected-to-detected ratio (*A*/*D*) of 48 at saturation. Also the percentage of affected population at the start (on the $18{\text {th}}$ day) is very small. These numbers are intuitively more satisfactory, and we now compare them with a serological study.

We could not find any nationwide serological study for Italy. A big serological study in Italy was carried out in the northeastern region of the country, which is one of the most affected regions in Italy, between May 5 and May 15^27^. It included approximately 6000 participants. This study reported a seroprevalence of $23.1\%(95\%\,\, \text{ CI } \,\, 22.0-24.1)$, while our continuum model predicts that $16.79\%$ and $17.74\%$ of the Italian population was infected on May 5 and May 15, respectively. Note that we have used the nation-wide data to fit the continuum model, while the serological study was carried out in a region with high incidence. It is not surprising then that our continuum model gives a lower estimate.

A small-*f* expansion of Eq. (13) with $2<m<3$, yields terms of the form $f^{3-m}$. This fractional power makes linear stability analysis impossible, but significantly improves the fit in the early stages of the pandemic, as shown in the supplementary material. Thus our fitted value of $m=2.6688$ for Italy provides indirect evidence for a fat tail in the distribution of *u*, which corresponds to an active role of superspreaders in the progression of the pandemic.

For distributions with faster decaying tails, or with no tails at all (i.e., with finite support), the dominant term on the right hand side of Eq. (13) turns out to be ${\mathcal {O}}(f)$, for small *f*. Linearization is then possible but as explained in the supplementary material, the fit in the initial stages is visibly poorer. One consequence of $m<3$ is that the idea of the $R_0$ is not applicable to this model. Note that as soon as the pandemic progresses even a little bit, and *f* takes a strictly positive value, *G* of Eqs. (14) and (15) has an exponentially decaying envelope, and the subsequent dynamics is better behaved. This is why in the latter stages of the pandemic, all the distributions studied in the supplementary material give equally good fits. A detailed theoretical investigation of the consequence of $m<3$ is left for future work. Here we restrict ourselves to noting from numerical simulations that good fits require $m<3$, i.e., a fat tail in the distribution of *u*.

Upon inspecting the local minima obtained from the fminsearch runs, we found that all the minima corresponding to low values of *E* have nearly identical values of *a*, *m* and $f_0$ (reported in the first row of Table 4) but different values of *p* and $\tau$. These observations are similar to those from the mean-$\beta$ model. Moreover, the values of *p* are low, while the values of $\tau$ vary over its entire assumed range. For more insight, we fix *a*, *m* and $f_0$, and plot $E_{0}$ in the $p-\tau$ plane (for low values of *p*) as shown in the mid-right panel of Fig. 1. We see that the lowest values of $E_{0}$ are obtained on a thin band cutting across the $p-\tau$ plane, spanning the entire assumed range of $\tau$ and a relatively much smaller range of *p*. Similar to the estimation results of the mean-$\beta$ model, $\tau$ and *p* remain indeterminate even for the continuum model. Upon plotting $E_0$ in the ${\bar{p}}-\tau$ plane in the bottom-right panel of Fig. 1, we observe that the thin band of minimum values corresponds to an almost fixed value of ${\bar{p}}\approx 0.0207$.

In the next section, we report results for Germany, the UK, and Spain. We will see that the main features of the results reported for the case of Italy hold for these countries as well.

## The cases of Germany, UK, and Spain

**Figure 3:**
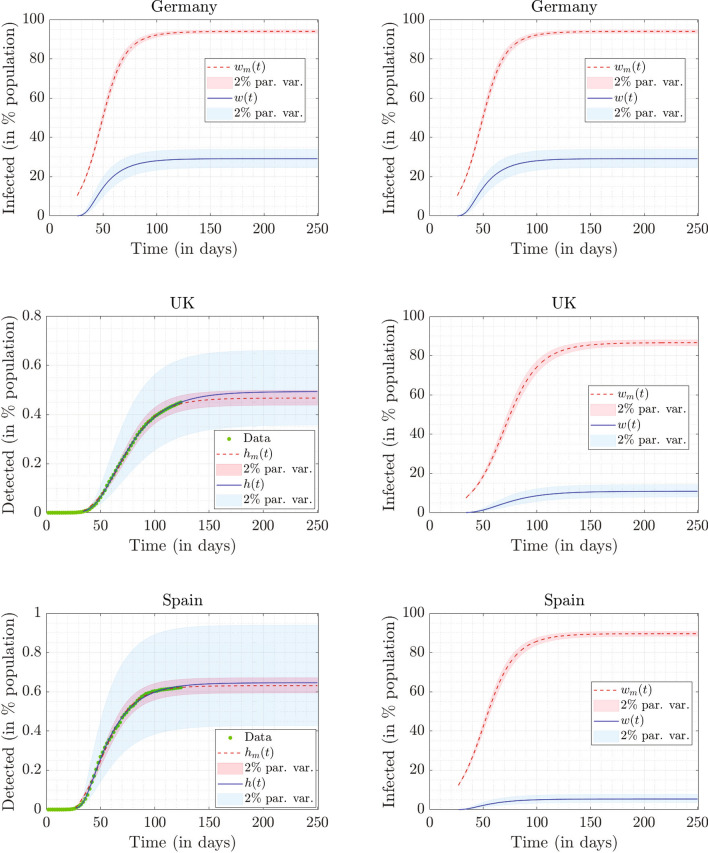
Top row: Fitted results for Germany, $h_m(250)=0.2206\%$, $h(250)=0.2246\%$, $w_m(250)=93.9666\%$ and $w(250)=29.1509\%$. Middle row: Fitted results for the UK, $h_m(250)=0.4671\%$, $h(250)=0.4934\%$, $w_m(250)=86.6384\%$ and $w(250)=10.9269\%$. Bottom row: Fitted results for Spain, $h_m(250)=0.6311\%$, $h(250)=0.6456\%$, $w_m(250)=89.6423\%$ and $w(250)=5.3903\%$. Data in percentage of population for detected cases, obtained from Worldometer, is plotted using green circles. We have plotted the data of alternate days for clarity. The fits obtained from the mean-$\beta$ model are shown using dashed red curves, while those from the continuum model are shown using solid blue curves. The parameters used in the mean-$\beta$ model and continuum model for obtaining the fit are shown in Tables 3 and 4, respectively. The red and blue shaded bands in all the figures correspond to $\pm 2\%$ variations in the parameters in the mean-$\beta$ model and the continuum model, respectively.

**Figure 4:**
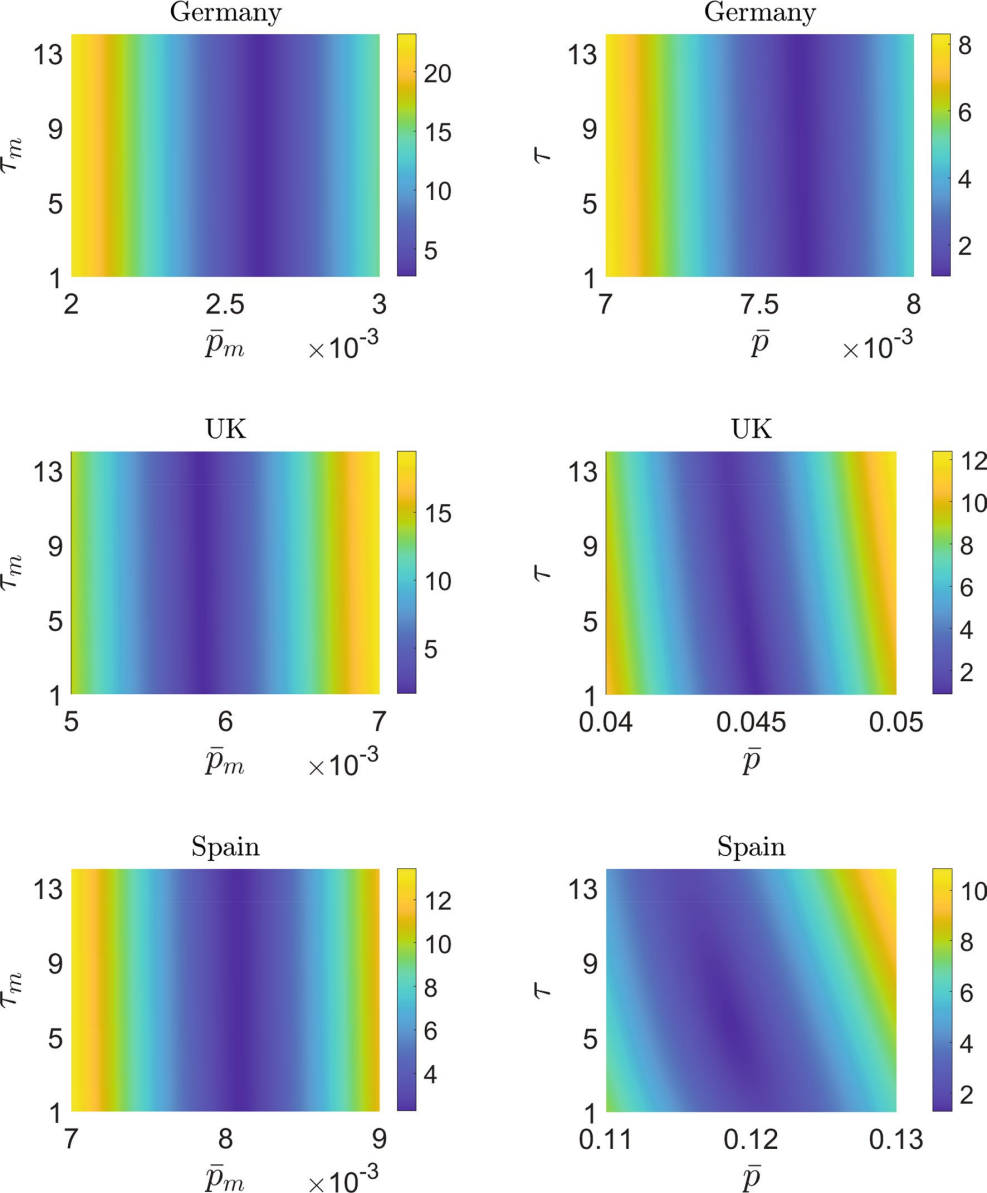
The left side shows the variation of $E_{0m}$ in the ${\bar{p}}_m-\tau _m$ plane (for low values of ${\bar{p}}_m$) obtained using the mean-$\beta$ model. The parameters $\beta _m$ and $V_0$ are fixed at the values reported in Table 3. The right side shows the variation of $E_{0}$ in the ${\bar{p}}-\tau$ plane (for low values of ${\bar{p}}$) obtained from the continuum model. The parameters *a*, *m*, and $f_0$ are fixed at the values reported in Table 4.

The best fits obtained using the two models for these three countries are presented in Fig. 3. For Germany (see the top-left panel of Fig. 3), the continuum model under-predicts and for Spain (see the bottom-left panel of Fig. 3), the continuum model over-predicts the actual data near the end. However, for the UK (see the mid-left panel of Fig. 3), the fit is excellent. The variation of $E_{0m}$ obtained from the mean-$\beta$ model in the ${\bar{p}}_m-\tau _m$ plane (with $\beta _m$ and $V_0$ fixed at values reported in Table 3) is plotted on the left panels of Fig. 4. The variation of $E_{0}$ obtained from the continuum model in the ${\bar{p}}-\tau$ plane (with *a*, *m* and $f_0$ fixed at values reported in Table 4) is plotted on the right panels of Fig. 4. The affected-to-detected (*A*/*D*) ratio corresponding to the best fits for the mean-$\beta$ model and the continuum model are reported in Tables 3 and 4, respectively.

We see that the fitting results are qualitatively similar to those obtained for Italy. However, there are a few observations that stand out in the results for the continuum model as highlighted below:

- We see in Table 4 that the optimum value of *m* for Germany, the UK, and Spain is around 2.5. This indicates that the infectivity distribution $\phi (\beta )$ for each of these countries has a fat tail as can be in Fig. 2. We see from the figure that most of the population has small infectivity (low value of $\beta$). However, these curves decay to zero very slowly, since the first moment of $\phi (\beta )$ is infinite, i.e.
  $$\begin{aligned} \int _{\beta =0}^{\infty } \beta \phi (\beta ) \, d\beta = \infty . \end{aligned}$$
  Such a distribution is consistent with the presence of ‘super-spreaders’ (people who have, or events that lead to, a high value of $\beta$). Our results are in line with findings of Wong et al.^28^ that the distribution of infections caused by an index case is fat-tailed. The role played by different kinds of super-spreading events, and empirical evidence for their role in the COVID-19 pandemic, are discussed in detail by Althouse et al.^29^ (see also references therein). See the works by Adam et al.^30^ and Britton et al.^31^ for mathematical models discussing the role of super-spreading in COVID-19. Super-spreading events have been widely reported for COVID-19 in the medical literature as well^32,33^.
- We see from Table 4 that the affected-to-detected ratio (*A*/*D*) is high for all the countries and varies between 8 for Spain and 130 for Germany.
- The ratio of symptomatic cases to detected cases can be approximated from the value of *p*. Symptomatic cases outnumber the reported cases by about 5 times in Spain, 12 times in the UK, 25 times in Italy, and 60 times in Germany.
- The serological study by Pollán et al.^8^ in Spain was carried out between April 27 and May 11, and about 61,000 people participated in it. It reports a seroprevalence of $5.0\%(4.7-5.4)$ by the point-of-care test and $4.6\%(4.3-5.0)$ by immunoassay. Our continuum model predicts that $4.04\%$ and $4.65\%$ of the Spanish population was infected on April 27 and May 11, respectively. In the UK, $6.8\%(5.2-8.6)$ of the population was affected by COVID-19 as of May 24^34^. Our continuum model predicts this number to be $8.63\%$. We could not find a comparable nationwide study for Germany. Several studies are underway, but their results are not as yet published^35^. However, the match with limited studies^36,37^ in Germany is poor, possibly because Germany’s partial lockdown approach requires different modeling, e.g., a model with spatial structure.

## Conclusions

In this work, we fit the data for the total number of infected people in four western European countries. We use two limiting cases of the time-delayed network SEIQR model: the mean-$\beta$ model and the continuum model. In earlier works, it was shown that for fast pandemics, each of these two models reduces to one non-linear delay differential equation.

After fixing the values of the biological parameters $\sigma$ and $\gamma$, we need to identify four parameters in the mean-$\beta$ model and five parameters in the continuum model. In both the cases, we see that there are many parameter sets that minimize the fitting error, yielding almost identical values of the objective function. All these sets have almost identical values of all parameters other than *p* and $\tau$. Other subsidiary quantities such as the total number of infected people, the affected-to-detected ratio, and the basic reproduction number are also close to each other in value. By plotting the fitting error in the $p-\tau$ plane (with other parameters fixed at their identified values), we see that a narrow band yielding minimum error cuts across this plane, spanning the entire assumed range of $\tau$ and a small range of *p* comprising low values. In this light, the $\tau$ values reported in Tables 3 and 4 may not be reliable.

We see from the results that the continuum model yields superior fits in comparison to the mean-$\beta$ model. Even the worst fit obtained from the continuum model, for Spain, has 2-norm fitting error of only $1.28\%$. Moreover, it gives reasonable and physically realizable values for all the epidemiological quantities. We conclude from the results that the predicted number of infected people depends on the assumed distribution of the infectivity rate. A single homogeneous infectivity rate overestimates the seroprevalence in the countries examined.

Both the models are relatively insensitive to small changes in the input parameters. The continuum model is more sensitive than the mean-$\beta$ model, which indicates indirectly that its parameter estimates are somewhat more robust.

The most important prediction from the models is that the total number of affected people far outnumber the people detected with COVID-19 in all the four countries. The continuum model predicts that the affected-to-detected ratio, in increasing order, is 8, 22, 48, and 130 for Spain, the UK, Italy, and Germany, respectively. The first three of these numbers are consistent with the serological surveys conducted in the corresponding countries. In particular, our estimated number of infected people in Spain in early May was about $5\%$ as per our model, in agreement with a nation-wide seroprevalence study^8^. We emphasize that the detailed work done in this seroprevalence study retains primary importance. Our work provides a mathematical supporting view of consistency, and is not intended to replace such detailed seroprevalence studies. Our numbers for affected people in Italy and the UK match reasonably well with seroprevalence data from these countries^27,34^. In contrast however, our predicted numbers for Germany are too high. This may be because the partial lockdown approach of Germany requires spatial structure within the model.

## Supplementary Information

Supplementary Information.

## Data Availability

All the calculations were done in Matlab. The codes and the data can be found at: https://doi.org/10.5281/zenodo.4419975.
