# Supplementary material for: Data suggest COVID-19 affected numbers greatly exceeded detected numbers, in four European countries, as per a delayed SEIQR model
Source: Sci Rep. 2021 Apr 14;11:8106. doi: 10.1038/s41598-021-87630-z (PMC8046823; doi:10.1038/s41598-021-87630-z)
Supplement: Supplementary file 1 — Supplementary Information. [file 41598_2021_87630_MOESM1_ESM.pdf]

Supplementary material for *Data suggest COVID-19 affected numbers greatly exceeded detected numbers, in four European countries, as per a delayed SEIQR model*

Sankalp Tiwari* C. P. Vyasarayani † Anindya Chatterjee‡

January 1, 2021

Outline

In the first section, we try different infectivity distributions in the continuum model, and conclude that the generalized Pareto distribution with two free parameters gives the best results. In the second section, we investigate the sensitivity of the mean-β model and the continuum model to the externally specified parameters σ and γ. To this end, we vary these parameters by $\pm 2\%$ around their 'nominal' values, and see that both the models are robust to small variations in these parameters.

1 Fits with other distributions in the continuum model

In the main paper, we used the generalized Pareto distribution for $\psi(u)$, which is related to the infectivity distribution in the population $\phi(\beta)$ as

$$\phi(u^2) = \frac{\psi(u)}{2u}. \tag{1}$$

We saw that the optimum fits were obtained for $\phi(\beta)$ with fat tails.

In this section, we try three other distributions for ψ: a modified exponential distribution, the generalized Pareto distribution with $m = 5$, and the beta distribution. These distributions are chosen for their variety. The modified exponential distribution

$$\psi(u) = \begin{cases} \frac{\lambda_1}{\lambda_1\lambda_2+1}, & \text{for } 0 \le u < \lambda_2 \\ \frac{\lambda_1}{\lambda_1\lambda_2+1} e^{-\lambda_1(u-\lambda_2)}, & \text{for } \lambda_2 \le u < \infty \end{cases}$$

has two free parameters (λ_1, λ_2), infinite support, and all moments finite. The generalized Pareto distribution, with $m = 5$,

$$\psi(u) = \frac{4a^4}{(a+u)^5}$$

has one free parameter (a), infinite support, and three finite moments. The beta distribution

$$\psi(u) = \begin{cases} \frac{u^{\lambda_1-1}(1-u)^{\lambda_2-1}}{\int_0^1 v^{\lambda_1-1}(1-v)^{\lambda_2-1}\,dv}, & \text{for } 0 \le u \le 1 \\ 0, & \text{otherwise,} \end{cases}$$

has two free parameters (λ_1, λ_2), finite support, and all moments finite.

*sankalpt@iitk.ac.in, snklptwr@gmail.com
†vcprakash@mae.iith.ac.in
‡anindya@iitk.ac.in, anindya100@gmail.com

The optimum fits obtained for the above distributions are plotted in Figures 1-4, for Italy, Germany, the UK, and Spain, respectively. For comparison, we also plot the optimum fit obtained using the generalized Pareto distribution with two free parameters in these figures. We see that for all the four countries, the two parameter generalized Pareto distribution fits the best, especially in the initial period. The zoomed-in views plotted in the right hand side panels of Figures 1-4 demonstrate the superiority of the two parameter generalized Pareto distribution in the initial period clearly. Fitting error (E_0) values attest to this; see Table 1 for a summary of the results from all the distributions. We also report the saturation value of percentage population infected in the last column of Table 1. The two parameter generalized Pareto distribution is in the best agreement with the results of serological surveys, and the other models clearly overestimate this number.

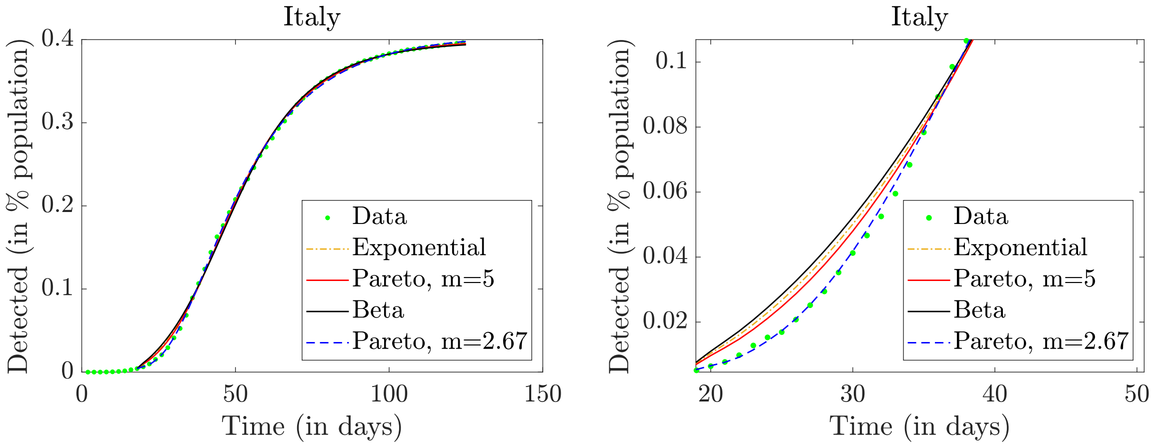

(a) (b)

Figure 1: Fitted results for percentage of detected population in Italy with different distributions. The figure on the right hand side shows the zoomed-in view of the initial period from the figure on the left hand side.

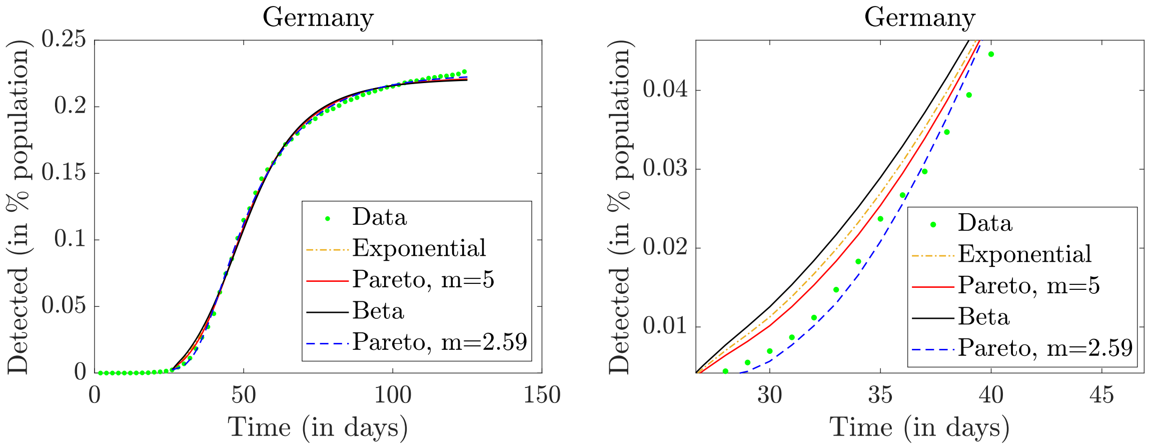

(a) (b)

Figure 2: Fitted results for percentage of detected population in Germany with different distributions. The figure on the right hand side shows the zoomed-in view of the initial period from the figure on the left hand side.

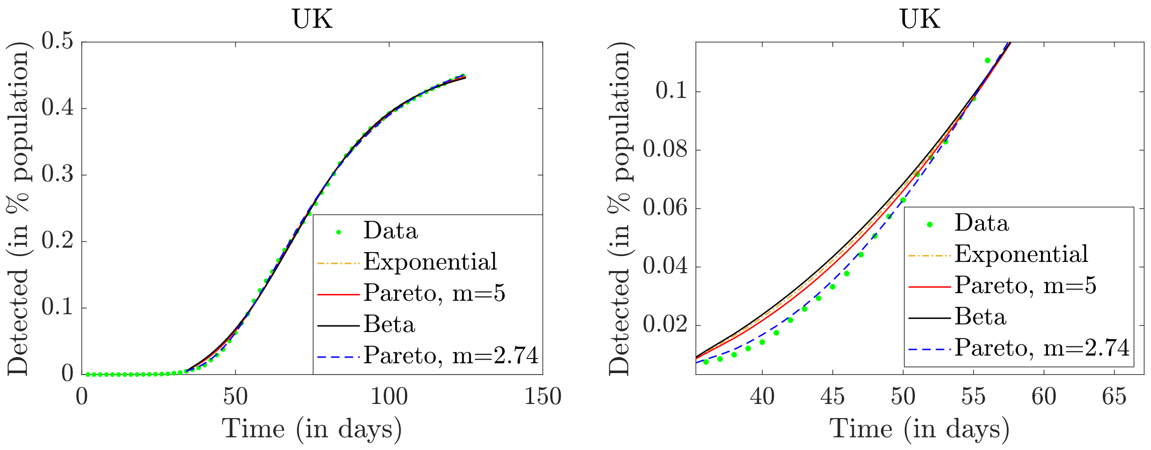

(a) (b)

Figure 3: Fitted results for percentage of detected population in the UK with different distributions. The figure on the right hand side shows the zoomed-in view of the initial period from the figure on the left hand side.

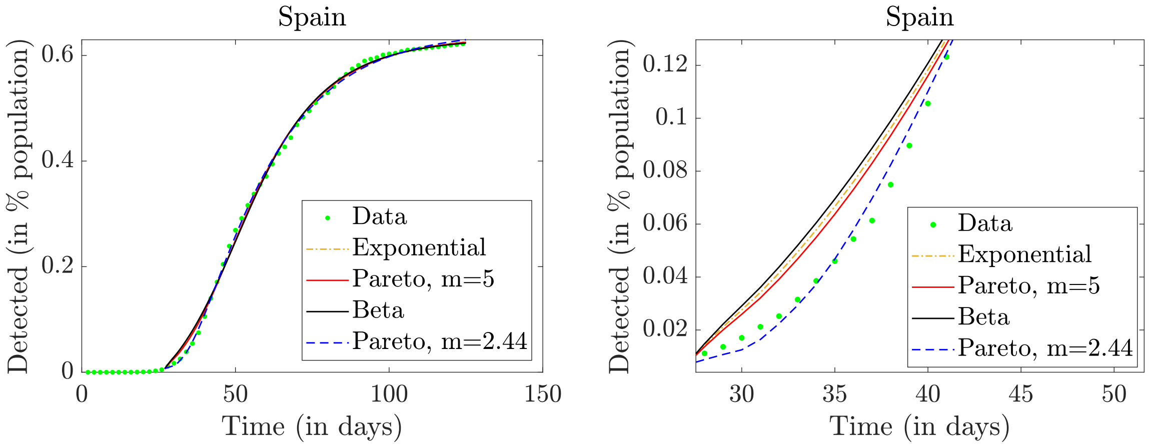

(a) (b)

Figure 4: Fitted results for percentage of detected population in Spain with different distributions. The figure on the right hand side shows the zoomed-in view of the initial period from the figure on the left hand side.

2 Sensitivity of the fitted parameters to the fixed parameters σ and γ

In this section, we test the robustness of the fitted parameters in the mean-β model and the continuum model with regard to small uncertainties in estimation of the fixed parameters σ and γ. To this end, we vary σ and γ by $\pm 2\%$ around their *nominal* values of 3 and 0.07, respectively. For the four combinations thus obtained, we compute the fitted parameters and other subsidiary quantities. These results are reported in Tables 2 and 3. We see that the fitted parameters and associated subsidiary quantities are relatively insensitive to small variations in σ and γ. The estimate of the A/D ratio is more sensitive to variations in σ and γ in the continuum model in comparison to the mean-β model; for instance, it varies by about 15% for Spain for the former as opposed to about 0.4% for the latter.

Table 1: Fitting results for detected population with different distributions.

Country	Distribution	Optimum parameters	E_0	Infected population (in %)
Italy	Generalized Pareto	$a = 0.1351$, $m = 2.6688$	0.6800	19
	Exponential	$\lambda_1 = 2.9483$, $\lambda_2 = 0$	1.4027	43
	Generalized Pareto ($m = 5$)	$a = 0.9241$	1.1688	47
	Beta	$\lambda_1 = 1$, $\lambda_2 = 1.6822$	1.6265	69
Germany	Generalized Pareto	$a = 0.1503$, $m = 2.5882$	1.0597	29
	Exponential	$\lambda_1 = 2.5853$, $\lambda_2 = 0$	1.8801	35
	Generalized Pareto ($m = 5$)	$a = 1.0856$	1.5928	57
	Beta	$\lambda_1 = 1$, $\lambda_2 = 1.4036$	2.2580	76
UK	Generalized Pareto	$a = 0.1185$, $m = 2.7392$	0.8896	11
	Exponential	$\lambda_1 = 3.2558$, $\lambda_2 = 0$	1.3379	50
	Generalized Pareto ($m = 5$)	$a = 0.8126$	1.1809	38
	Beta	$\lambda_1 = 1$, $\lambda_2 = 1.9204$	1.4657	62
Spain	Generalized Pareto	$a = 0.0527$, $m = 2.4357$	1.2844	5
	Exponential	$\lambda_1 = 2.9699$, $\lambda_2 = 0$	1.9621	44
	Generalized Pareto ($m = 5$)	$a = 0.9202$	1.8168	47
	Beta	$\lambda_1 = 0$, $\lambda_2 = 1.7130$	2.1018	68

Table 2: Sensitivity of the mean-β model to $\pm 2\%$ variation in σ and γ.

Country	β_m	$\bar{p}_m$	V_0	E_{0m}	A/D	R_0
Italy	0.182 ± 0.002	$0.0048 \pm 1.86 \times 10^{-5}$	0.486 ± 0.007	1.877 ± 0.009	228 ± 0.95	2.596 ± 0.034
Germany	0.210 ± 0.003	$0.0026 \pm 6.04 \times 10^{-6}$	0.521 ± 0.007	2.683 ± 0.013	426 ± 1.22	2.989 ± 0.041
UK	0.164 ± 0.002	$0.0058 \pm 3.09 \times 10^{-5}$	0.477 ± 0.007	1.621 ± 0.007	185 ± 1.00	2.324 ± 0.029
Spain	0.179 ± 0.002	$0.0081 \pm 3.27 \times 10^{-5}$	0.739 ± 0.010	2.248 ± 0.007	142 ± 0.62	2.530 ± 0.032

Table 3: Sensitivity of the continuum model to $\pm 2\%$ variation in σ and γ.

Country	a	m	$\bar{p}$	f_0	E_0	A/D
Italy	0.135 ± 0.007	2.669 ± 0.017	0.0208 ± 0.0014	0.0020 ± 0.0003	0.680 ± 0.008	48 ± 3.30
Germany	0.151 ± 0.009	2.590 ± 0.022	0.0076 ± 0.0005	0.0008 ± 0.0002	1.059 ± 0.017	130 ± 8.13
UK	0.118 ± 0.001	2.739 ± 0.004	0.0450 ± 0.0020	0.0048 ± 0.0001	0.890 ± 0.004	22 ± 0.97
Spain	0.051 ± 0.002	2.433 ± 0.007	0.1294 ± 0.0196	$0.0002 \pm 2.2134 \times 10^{-5}$	1.284 ± 0.005	8 ± 1.19
